# Patients’ experiences of analgesic use for chronic musculoskeletal pain in primary care: An interview study in Germany

**DOI:** 10.1080/13814788.2026.2747452

**Published:** 2026-11-02

**Authors:** Nele Kornder, Sophia Naomi Groffebert, Victoria Jessica Hill, Annette Becker, Annika Viniol, Nicole Lindner

**Affiliations:** Department of Primary Care, Marburg University, Marburg, Germany

**Keywords:** Musculoskeletal pain, general practitioners, analgesic prescribing, shared decision-making

## Abstract

**Background:**

Chronic musculoskeletal pain is a leading cause of primary care consultations and presents challenges for analgesic prescribing across health systems. While pharmacological treatments are widely used, little is known about patients’ experiences with non-opioid analgesics and their preferences for involvement in treatment decision-making.

**Methods:**

This qualitative study used semi-structured interviews with 17 patients with chronic musculoskeletal pain recruited from primary care practices in Germany. Interviews were audio-recorded, transcribed verbatim, and analysed thematically following Braun and Clarke’s approach using a combined inductive–deductive coding strategy. Coding was conducted by two researchers with iterative consensus discussions. A patient advisory board contributed to study design and interpretation.

**Results:**

Analgesics were described as situational adjuncts rather than standalone treatments, with non-pharmacological strategies often prioritised. Participants expressed three orientations towards analgesics: accepting, cautious and explicitly multimodal. Non-opioid analgesics – particularly metamizole – were commonly used, while opioids played a minor role. Patients’ decision-making processes ranged from autonomous to delegated and were shaped by trust in clinicians and symptom burden. Information about risks was inconsistently reported, and continuity of care was central to confidence in analgesic use.

**Conclusion:**

Analgesic use in chronic musculoskeletal pain is shaped by dynamic decision-making processes, trust and continuity of care. These findings highlight the importance of clear risk communication and relational continuity in supporting patient-centred analgesic management in primary care.

## KEY MESSAGES

- Analgesics are used as situational tools within broader self-management strategies rather than as standalone treatments.
- Decision-making is dynamic and shaped by trust, continuity of care and symptom burden, requiring flexible GP communication.
- Deprescribing and medication review remain under-addressed and should be integrated proactively into routine primary care.

## Introduction

Musculoskeletal pain represents one of the largest contributors to disability worldwide and is a leading cause of consultations in primary care [1,2]. Management of chronic musculoskeletal pain is complex, often requiring long-term follow-up, repeated treatment adjustments and sustained primary care support [3]. Within this complexity, pharmacological pain treatments form an important, but not exclusive, component of care alongside non-pharmacological approaches [4]. Decisions about analgesic use are central to clinical practice, requiring clinicians to balance benefits, risks and patient preferences in routine care.

In the United States, pharmacological treatment – especially opioids – is frequently used and often takes precedence over non-drug interventions such as physiotherapy [4]. In contrast, German drug-utilisation data indicate that opioid prescribing for non-cancer pain has declined over the past 15 years, with no evidence of an opioid crisis comparable to that reported in North America [5]. Over the same period, overall analgesic use has increased, driven primarily by non-opioid agents [6]. A distinctive feature of analgesic care in Germany is the widespread, yet regionally variable, use of metamizole [7], a non-opioid analgesic that is unavailable or severely restricted in several countries because of the rare risk of agranulocytosis. International data further demonstrate substantial cross-national variation, with generally lower opioid prescribing across Europe than in North America [8,9]. While the availability of metamizole may contribute to differences in prescribing patterns, lower opioid prescribing in Germany is likely to reflect multiple factors, including national prescribing practices and guideline recommendations.

Most qualitative research on primary care pain management has focused on opioid prescribing [10,11], despite non-opioid analgesics accounting for a substantial share of treatment [7]. Qualitative evidence capturing patients’ experiences across the full spectrum of analgesic use in chronic musculoskeletal pain remains limited. In a previous qualitative study, GPs described prescribing as a complex process. They reported favouring non-opioid agents such as metamizole and NSAIDs over opioids, embedding medication within multimodal strategies, and emphasising shared decision-making and strong doctor–patient relationships, while also highlighting challenges related to psychosocial factors and patient expectations [12].

Given this background, this study aimed to explore patients’ experiences of analgesic use for chronic musculoskeletal pain in primary care, focusing on expectations, decision-making and perceived support needs to inform patient-centred care.

## Methods

### Study design

We conducted a semi-structured interview study with individuals experiencing chronic musculoskeletal pain to explore their perspectives on the use of analgesics in general practice. A qualitative approach was chosen to capture complex decision-making, patients’ experienced information and support needs. Written informed consent was obtained from all participants following a detailed explanation of the study’s aims and procedures, in accordance with the principles of the Declaration of Helsinki. The study was approved by the Ethics Committee of the University of Marburg (ethics approval ID: 24-76-BO).

A patient advisory board contributed to the development of the interview guide by reviewing its content and suggesting additional topics relevant to patients’ experiences of primary care pain management, which were incorporated into the final guide. Preliminary findings were subsequently presented to the patient advisory board for feedback on their relevance and resonance from a patient perspective.

The research team consisted of two medical doctoral candidates (SG, VH) and four academic and practising GPs with experience in qualitative research (NK, NL, AB, AV). The researchers reflected on their clinical and personal experiences with musculoskeletal pain and analgesic use throughout the research process to enhance reflexivity and transparency.

### Study sample

Participants were adult patients with chronic (defined as lasting longer than three months [13]), non-malignant musculoskeletal pain who self-reported having consulted their GP regarding pain-related issues within the previous six months.

Exclusion criteria included cognitive impairment or insufficient German language proficiency to provide informed consent.

Recruitment took place in the German federal states of Hesse, Bavaria and Baden-Württemberg using purposive sampling to achieve variation in participant characteristics and analgesic experiences. Potential participants were informed about the study through posters and flyers displayed in general practices, hospitals and other community settings (including self-help groups). GPs could also inform potentially eligible patients about the study but did not select participants. Interested patients contacted the research team directly or agreed to have their contact details forwarded by the practice.

Participants received written study information, a consent form and a short sociodemographic questionnaire, which included the German version of the Autonomy Preference Index (API) [14], a validated questionnaire assessing patients’ preferences for information and participation in medical decision-making. Recruitment of participants was guided by the concept of information power [15]. Given the study’s focused research aim, the specific participant group with direct experience of long-term analgesic use, and the rich data generated through in-depth interviews, the sample was considered to provide sufficient information power to address the research question.

### Data collection

Semi-structured interviews were conducted between November 2024 and December 2025 by the two female medical doctoral candidates (SG, VH). SG and VH were trained in qualitative interviewing, including participation in a training course, and received ongoing feedback from NK and NL. Participants chose whether the interview was held face-to-face at their home, the recruiting general practice, the Institute of General Practice in Marburg or in hospital facilities, by telephone or via video conference (BigBlueButton). Field notes were taken to complement the recordings.

The interview guide was informed by a prior qualitative study with GPs [12] and refined with input from the patient advisory board and the research team. It focused on patients’ experiences with the use of analgesics for musculoskeletal pain, their perceptions of communication and shared decision-making in general practice, and their needs and expectations regarding pain management (see Table 1).

**Table 1.** Interview guide used for semi-structured interviews.

Main topic	Sample question
1. Personal approach to dealing with pain	How do you deal with pain in your everyday life?What do you do when you are in pain?
2. Pain experience in the last six months	Have you had musculoskeletal pain in the last six months?Since when and where?
3. Expectations, reasons and preparations for the last GP visit	What expectations did you have for your doctor’s appointment?Do you gather knowledge in advance (for example, search online about symptoms or treatment options)?
4. Quality of the GP–patient relationship	How long have you been a patient at this GP practice?
5. Addressing pain medication during the GP consultation	What did the doctor say about pain medication?Were side effects and interactions with other medications discussed?
6. Patients’ experiences with musculoskeletal pain treatment	To what extent did you feel your needs were taken into account regarding medication-based pain management?To what extent did your GP take enough time for you?
7. Collaboration between GPs and other medical specialties	How do you feel about the collaboration between GPs and other medical specialties?Do you believe it has an impact on your treatment?
8. Personal views on pain medication and experiences with it	What is your general view on pain medication?Are you more open to pain medication, or would you prefer to try non-medication options first?
9. Need for support in musculoskeletal pain therapy	Do you see a need for support regarding musculoskeletal pain therapy?What form could this take?From your point of view, what would an ideal consultation for musculoskeletal pain and painkillers look like?
10. Therapy decision-making preferences	Do you generally want to participate in deciding on the treatment strategy, or should your doctor make decisions alone?

Abbreviations: GP = general practitioner.

The table outlines the main topics and example questions used to guide the interviews with participants.

### Data analysis

All interviews were audio-recorded, transcribed verbatim and pseudonymised. The dataset was managed in MAXQDA 24 (VERBI Software). Quotations were translated into English for publication with AI assistance (ChatGPT) and verified for accuracy.

The study was underpinned by reflexive thematic analysis following Braun and Clarke’s six-step approach, using a combined deductive–inductive coding strategy [16,17]. Two researchers (SG, VH) familiarised themselves with the transcripts, took analytical notes, and independently coded a subset of transcripts to develop a preliminary framework. This framework was iteratively refined through team discussions (NK, NL) and applied to all transcripts. Codes were then organised into themes and subthemes, and their interrelationships were mapped to capture overarching patterns. The final thematic structure was developed collaboratively to ensure analytic rigour and transparency. The study process underwent internal peer review within the research group and was presented at the German GP Conference for external feedback. Sociodemographic data and API scores [14] were analysed descriptively. ChatGPT (OpenAI) was used for language editing.

## Results

We interviewed 17 individuals with chronic musculoskeletal pain (10 women, 7 men). The majority of interviews were conducted in person (*n* = 9 in person, *n* = 7 via telephone, *n* = 1 via video conference). Five individuals were excluded during screening for not meeting inclusion criteria, and nine did not proceed to interview after initial contact. No interviews were discontinued. Interview duration ranged from 25 to 63 min (mean = 43 min). Participants’ ages ranged from 39 to 82 years (mean = 62.88). Pain sites were heterogeneous (back pain, upper/lower extremity pain, and widespread musculoskeletal pain). API scores indicated low decision-involvement preference (median 25.00, range: 0.00–100.00) and high information preference (median 96.43, range: 71.43–100.00). Characteristics of the interview partners are summarised in Table 2; the pain medications mentioned by participants are presented in Supplement 1.

**Table 2.** Characteristics of study participants.

Participant	Age (years)	Frequency of consultations with GP^a^	Body region of the pain^b^	API ^c^	
				Part.	Info.
P-01	66	Every 1–3 months	Not given	18.75	85.71
P-02	62	Every 1–3 months	Back and lower extremity	50.00	100.00
P-03	59	Every 1–3 months	Lower extremity	6.25	100.00
P-04	46	Every 1–3 months	Back, upper extremity and lower extremity	37.50	100.00
P-05	58	Every 1–3 months	Back and lower extremity	25.00	71.43
P-06	77	Weekly	Back	0.00	96.43
P-07	47	Weekly	Entire musculoskeletal system	68.75	89.29
P-08	80	Weekly	Back	0.00	92.86
P-09	60	Every 1–3 months	Back, upper extremity and lower extremity	18.75	100.00
P-10	82	Every 4–6 months	Entire musculoskeletal system	12.50	100.00
P-11	67	Every 1–3 months	Lower extremity	50.00	96.43
P-12	64	Weekly	Entire musculoskeletal system	12.50	85.71
P-13	39	Not given	Back and lower extremity	43.75	96.43
P-14	77	Every 1–3 months	Back	31.25	100.00
P-15	58	Every 1–3 months	Back and upper extremity	25.00	96.43
P-16	73	Every 1–3 months	Back and upper extremity	0.00	100.00
P-17	54	Every 1–3 months	Entire musculoskeletal system	100.00	100.00

^a^Consultations for musculoskeletal pain.

^b^Back pain includes spinal pain from neck to pelvis; upper extremity pain includes musculoskeletal pain of shoulders, elbows or hands; lower extremity pain includes musculoskeletal pain of hips, legs or knees.

^c^Transformed scale scores (range 0–100) according to the Autonomy Preference Index (API)-DM manual. Part. denotes the participation preference scale and Info. the information preference scale. Higher scores indicate stronger preference.

The table summarises demographic and clinical characteristics of the interviewed patients, including age, pain location, consultation frequency and decision-making preferences.

Thematic analysis identified six interrelated themes (see Figure 1):

**Figure 1.**
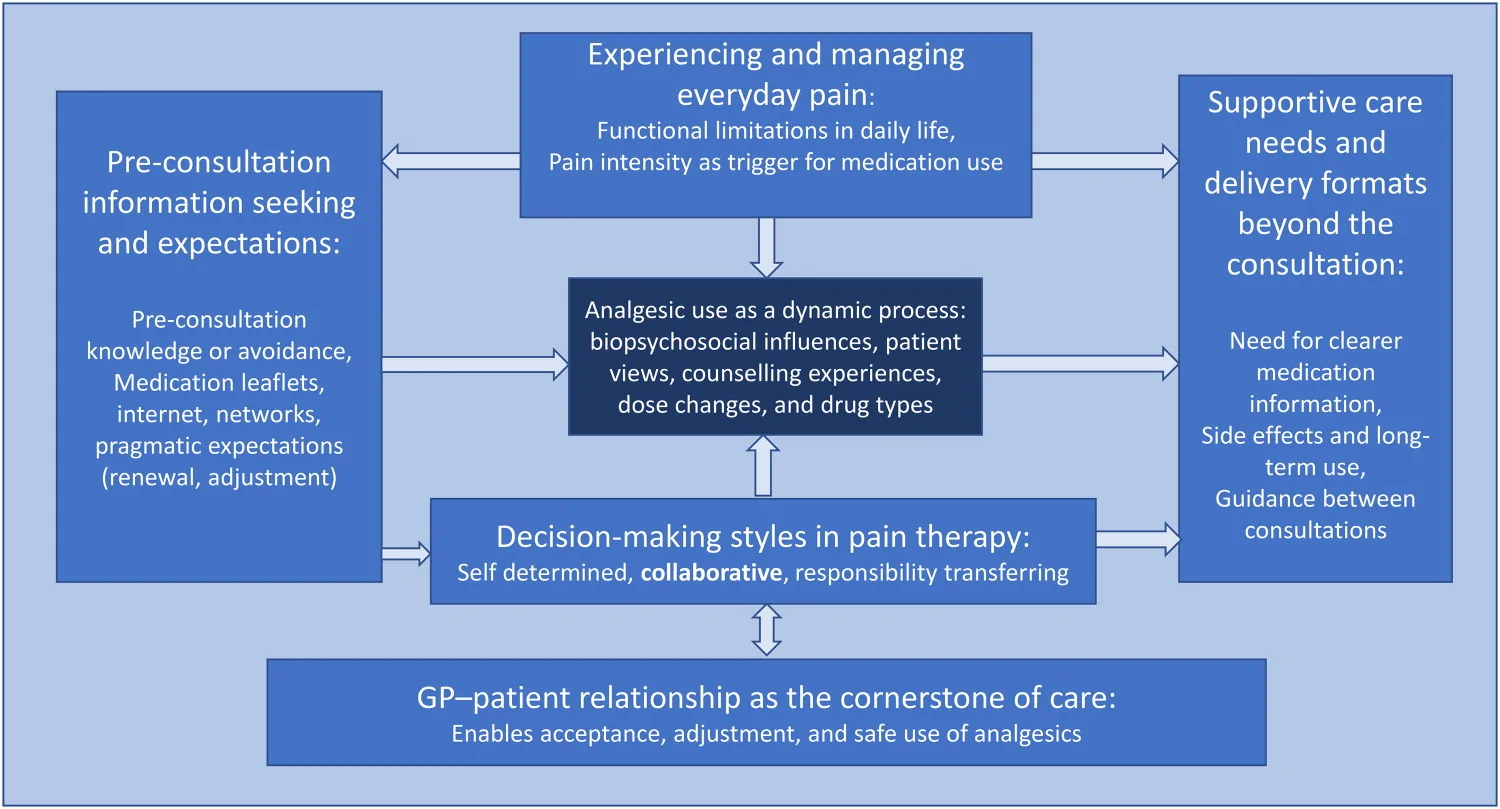
Overview of main themes of patients’ experiences with analgesic therapy in primary care. Six interrelated themes illustrate how everyday pain, pre-consultation expectations and support needs shape navigating analgesic use. The individual decision-making style and the GP–patient relationship underpins all themes.

1. Experiencing and managing everyday pain: perceptions, functional limitations, coping
2. Analgesic use as a dynamic process: biopsychosocial influences, patient views, counselling experiences, dose changes, drug types
3. Pre-consultation information seeking and expectations
4. Decision-making styles in pain therapy: autonomous, collaborative, delegated
5. The GP–patient relationship as a cornerstone of care
6. Supportive care needs and delivery formats beyond the consultation

### Experiencing and managing everyday pain: Perceptions, functional limitations, coping

Participants described their musculoskeletal pain as a constant and often fluctuating part of everyday life. Pain seemed to be influenced by factors such as activity, fatigue and, for some, highly individual environmental triggers such as weather changes:

This [pain] also changes … weather sensitivity plays a role too. (P-02)

Functional limitations were described as a central part of everyday life, affecting mobility, household activities, and employment. Restrictions in walking, climbing stairs, bending, or standing for prolonged periods were commonly reported. For some, these limitations led to substantial losses in independence and social participation:

I could no longer go to work … the pain got so bad that I couldn’t walk anymore. Even just standing hurt. (P-05)

To cope with these challenges, participants outlined a combination of pacing strategies, rest periods, non-pharmacological methods and, when necessary, analgesics. They described to balance these options according to perceived effectiveness, accessibility and personal experience:

If I’ve done something exhausting, I take a rest break … it works quite well. And I’ve learned to dose my work – what I once did in an hour, I now do in three stages. (P-05)

Some participants reported that over time, they had learned to manage their pain more effectively and relied less on medication, although this adaptation varied between individuals:

You can manage it better and reach for medication less often. (P-08)

Despite differing levels of impairment and adaptation, participants partly described considerable resilience, yet the burden of daily functional limitations remained a central part of their lived experience.

### Analgesic use as a dynamic process: Biopsychosocial influences, patient views, counselling experiences, dose changes, drug types

Within this theme the following sub-themes emerged: (1) pain management within a biopsychosocial context; (2) different underlying views on pain management; (3) experience and expectations of counselling about analgesics; (4) experience and expectations on reductions or discontinuation; and (5) experience with different types of analgesics.

(1) Pain management was described as a dynamic process in which pharmacological and non-pharmacological strategies were continuously weighed against each other and embedded within a broader biopsychosocial context. Participants emphasised that musculoskeletal pain affected not only physical functioning but also psychological well-being, with stress, mood and work-related demands shaping both pain experience and treatment decisions:

The psyche plays a big role. If a person says from the beginning, ‘I don’t feel like it, I’m not going to do it’, then the doctor will not be successful either. (P-05).

Overall, participants valued analgesic use being revisited and jointly evaluated in consultations, embedded within a broader therapeutic strategy that acknowledged psychosocial influences and avoided a purely prescription-focused approach:

Not just, ‘Here’s your prescription, see you next time’, and that’s it. (P-01)

(2) In our interviews, three underlying orientations towards pain medication emerged: i) a positive and accepting orientation, in which analgesics were valued as highly effective when needed:

Pain medication is one of humanity’s greatest inventions. (P-11).

ii) a sceptical or cautious orientation, characterised by concerns about polypharmacy and symptom-oriented treatment:

I’m not happy with so many medications … the pain goes away, but the cause isn’t treated. (P-02);

iii) a multimodal orientation, in which medication was deliberately positioned as a last resort within a broader strategy prioritising non-pharmacological approaches:

Before I take an ibuprofen, I first try conventional methods … I take the pill only when there’s no other option. (P-05).

(3) Counselling about analgesics typically emerged in response to symptom exacerbations, declining effectiveness or side effects and was initiated by both patients and GPs. However, experiences of counselling varied. Some participants reported minimal information and relied primarily on package leaflets:

No, I wasn’t told. I always just read the leaflet myself. (P-02)

Others described more detailed explanations regarding risks and precautions:

She really explained everything – the side effects, how they might show up. (P-06)

While information about dosing and side effects was sometimes mentioned, explanations of mechanisms of action were rarely reported.

(4) Regarding the experience on reductions or discontinuation analgesics, some participants reported to have attempted dose reductions or discontinuation – often motivated by side effects; others found that functioning without analgesics was difficult or impossible:

It was incredibly difficult to admit that without Tramadol and Novalgin I can’t walk anymore. (P-04)

(5) Looking closer at the experience with different types of analgesics, medication regimens seem to range from NSAIDs and, most commonly, metamizole to muscle relaxants, occasional cannabis-based therapies and less frequently opioids.

### Pre-consultation information seeking and expectations

Participants seem to vary widely in how they prepared for GP visits and what they expected from them. Some deliberately avoided online searches, since they felt it was confusing or anxiety-provoking:

I’m really against Dr Google … it only makes you worry. (P-01)

Others engaged in extensive pre-consultation information seeking, including patient forums, treatment options and rare conditions, sometimes using strategies to identify reliable sources:

I try to read only from serious websites … I even did a course for that. (P-07)

Some interview partners reviewed medication leaflets to prepare questions for their GP, whereas others relied on personal networks, such as medically trained friends or family.

One participant also described how they uncritically use newer digital tools, including generative AI, to inform themself and formulate concrete treatment expectations:

ChatGPT … I do that. And then I told my GP what I would like her to prescribe. (P-16, laughs)

### Decision-making styles in pain therapy: Autonomous, collaborative, delegated

Participants articulated markedly different preferences regarding their involvement in pain-related treatment decisions. These preferences formed a continuum between autonomy and delegation and seem to be shaped by prior illness experiences, trust in the GP and situational factors. Three decision-making orientations emerged: (1) *self-determined*, (2) *collaborative*, and (3) *responsibility-transferring.*

(1) Participants with a self-determined orientation emphasised autonomy, wishing to be fully informed while retaining the final say in treatment decisions.

I’ve already experienced so much that I want to decide this in consultation [with the GP]. It’s my body, it’s my life. (P-04)

(2) In our interviews the collaborative orientation framed decision-making as a shared process based on dialogue and mutual respect. Participants valued being asked for their views, having options explained and reaching agreements together.

Then I’m asked whether we should try it that way, and we agree that it fits for both of us. (P-09)

Several participants linked shared decision-making to better adherence, empowerment and therapeutic success, emphasising that treatments would more likely work when patients agree with the chosen approach:

I have to want it myself. Otherwise, it won’t help me. (P-11)

(3) Participants with a responsibility-transferring orientation preferred to delegate decisions to their GP once adequate explanations were provided. Trust in the GP’s expertise was central, and clear recommendations were welcomed rather than negotiated:

I want him [GP] to explain why I should take it. Then I’ll take it exactly as he says. He’s the expert, I’m the layperson. (P-08)

Overall, patients’ individual desired levels of involvement seem to vary considerably. Preferences could shift depending on health status, decisional capacity and the nature of the problem (e.g. stronger guidance in complex/overwhelming situations):

Shared would be ideal, but sometimes it’s better when the GP makes recommendations based on experience. (P-03)

### The GP–patient relationship as a cornerstone of care

Participants consistently emphasised the central role of their GP in analgesic management for chronic musculoskeletal pain. Many valued long-term continuity of care and the resulting sense of security.

GPs were partly viewed as the only clinicians with a holistic understanding of patients’ histories and broader health needs, in contrast to specialists’ narrower focus:

The GP is the one who knows me best and sees the whole picture. (P-04)

Adequate consultation time seem to be a key reason for trust and satisfaction. Many reported unusually long or flexible appointments – sometimes 20–30 min or even an hour – which seemed to enable a detailed consultation:

She [GP] takes half an hour – she wants no rush. (P-04)

Even when routine visits were brief, participants felt they could speak freely and receive more time whenever needed. Across interviews, patients described feeling understood, respected and taken seriously, which they saw as central to good care:

I feel 100% understood – completely supported. (P-03)

Several participants described larger group practices with frequently changing doctors as disrupting continuity and trust, reinforcing the value they placed on having a consistent personal GP.

### Supportive care needs and delivery formats beyond the consultation

Participants were generally satisfied with consultations about analgesics, and additional support needs were not consistently reported. However, some participants described unmet needs for structured support that extended beyond the consultation itself. They expected care to move beyond issuing prescriptions towards clearer guidance and contextualisation of treatment options.

A key gap concerned practical medication information, particularly regarding mechanisms of action, side effects and interactions, which patients linked to greater confidence and adherence.

Expectations also included better integration of non-pharmacological measures and coordinated referrals, while maintaining the GP as the central point of care.

Views on additional supportive tools were mixed. Some welcomed analogue or digital formats – such as telephone contact, printed materials, or apps for reporting pain and medication effects – to improve communication and reduce administrative burden.

If the doctor prescribes something, then they want to know whether it works or not? … This lack of therapy monitoring … can be mitigated through an app … Ideally, a multiple-choice system … How did it work? What is the pain intensity now? Has it decreased? … Do you want to see me [GP] again? (P-16)

Others perceived limited benefit, especially in the context of complex conditions or low digital affinity. Across accounts, supportive measures were expected to be accessible, complementary, and clearly secondary to personal contact.

## Discussion

### Summary

In our study, patients with chronic musculoskeletal pain described analgesic use as part of an ongoing balancing process rather than a standalone solution. Non-opioid analgesics, particularly metamizole, predominated. Trust, continuity and adaptable decision-making shaped confidence in medication use, while information gaps and limited structured review were identified as unmet needs.

### Comparison with literature

Participants’ descriptions of musculoskeletal pain as persistent yet fluctuating align with contemporary biopsychosocial models that conceptualise chronic pain as a dynamic process rather than a static symptom [18]. In line with this perspective, patients portrayed pain management as an ongoing effort to regulate activity, adapt routines and adjust medication in response to functional limitations. Qualitative syntheses similarly demonstrate that chronic musculoskeletal pain influences identity, everyday functioning and healthcare engagement, reinforcing the need to view pain as a lived experience beyond biomedical explanations [19].

Participants valued analgesics for symptom relief, yet dose adjustments were rarely raised explicitly within consultations, even when ambivalence was present. Instead, patients described informal strategies to limit medication use, such as symptom-based intake or combining pharmacological and non-pharmacological approaches. This mirrors qualitative evidence of cautious and pragmatic medicine-taking in chronic illness, characterised by self-regulation of regimens and limited discussion of modifications with clinicians [20].

In our study, a strong and trusting GP–patient relationship emerged as central in the context of analgesic use. Participants described continuity of care, sufficient consultation time and feeling taken seriously as enabling them to use pain medication in a considered and adaptive way. This is supported by the broader qualitative literature on chronic pain management, which consistently emphasises relational continuity, holistic understanding and the GP’s coordinating role as central to effective care [21–23]. Our findings extend this evidence by illustrating how these relational factors shape patients’ experiences and use of analgesics in primary care.

Taken together with our previous GP study – where many GPs described a collaborative approach, but some reported being cast primarily as service providers – our interview data suggest that decision-making styles appear to be situational and relational [12]. Patients shifted between autonomy, shared decision-making and delegation depending on symptom burden, treatment complexity and trust in their GP. Although participants generally expressed a high preference for receiving information on the API, this was not always reflected in their reported information-seeking behaviour. Consultations were often approached pragmatically and focused on specific concerns, such as prescription renewal, symptom relief or treatment adjustments, with the GP serving to validate or contextualise information obtained beforehand. In our sample, the API indicated a marked preference for being well informed alongside comparatively low preference for active participation in medical decisions. This pattern is consistent with the API’s distinction between information-seeking and decision-making autonomy and with earlier evidence that many patients want extensive information while still preferring physicians to take the lead in decisions, particularly as perceived illness severity increases [14]. Similar variability in decision-making preferences has been described in the context of deprescribing among older adults, where distinct patient types range from more active and questioning to more trusting and delegating approaches to medication decisions [24,25]. In our interviews, this tendency towards ‘informed delegation’ was particularly evident when symptom burden or treatment complexity increased.

Regional prescribing patterns contextualise our findings: in Germany, patients often receive metamizole rather than opioids for musculoskeletal pain, a practice not permitted in the UK. There, despite NICE guidance discouraging opioids for several chronic musculoskeletal pain conditions, routine British primary care data show that opioids remain commonly dispensed [13,26].

In our interviews, experiences of counselling about side effects varied. However, participants rarely mentioned discussion of potential adverse effects of metamizole such as agranulocytosis, whereas GPs in a preceding qualitative study reported routinely providing this information [12]. This discrepancy may reflect differences between intended counselling and perceived information in routine consultations. Additionally, patients seldom initiated dose reductions or discontinuation, and conversations about stopping medications were rarely raised, suggesting that active discussions about safely adjusting therapy are not routinely part of primary care interactions. This aligns with findings from a qualitative study of GPs using a decision-support tool for PPI deprescribing, where successful medication reduction depended less on the tool itself and more on established doctor–patient relationships and consultation context [27]. Yet, NICE [13] and German guidelines [28] encourage regular review of ongoing analgesic use and established deprescribing frameworks emphasise that medication withdrawal should be a structured, clinician-led process requiring proactive review and shared planning rather than occurring spontaneously [29]. Consistent with evidence on opioid therapy in chronic pain, our findings suggest that similar challenges exist for non-opioid analgesics, with patients receiving limited support for medication adjustment [30].

Future research should examine how deprescribing can be better integrated into routine primary care and explore whether the patient orientations identified in this study are transferable to other care contexts and larger populations.

### Strengths and limitations

This study provides qualitative insights into patient experiences of chronic musculoskeletal pain and decision-making around analgesics in German primary care, highlighting perspectives rarely captured in survey-based research. A key strength is the comprehensive view of the use of analgesics beyond opioids, which is particularly relevant given that opioids are generally discouraged in guidelines, yet remain in use. Trustworthiness was strengthened through involvement of multiple researchers, input from a patient advisory board throughout the study, and critical feedback from the wider research community.

However, some limitations should be considered. All participants and researchers were familiar with the German healthcare system, which may limit the transferability of findings to other healthcare contexts. Selection bias is possible, as participants were self-selected long-term patients with established GP relationships. Consequently, newer patients, those with lower continuity of care, and less engaged individuals are likely underrepresented. Furthermore, the use of different interview modes may have influenced participants’ responses and the dynamics of the interviews. Future research should examine how deprescribing can be better integrated into routine primary care and assess the distribution and relevance of the patient orientations identified in this study in larger populations using quantitative methods.

## Conclusion

This study showed that patients differ considerably in their orientations towards analgesic use and their preferences for involvement in treatment decisions. Trust, continuity of care and individualised communication emerged as central factors shaping analgesic management in primary care.

For practice, analgesic management should be understood as a process that is regularly revisited rather than a one-off decision. Proactive medication reviews, including explicit discussions of benefits, harms, ongoing need and opportunities for deprescribing, may support more patient-centred care.

## Supplementary Material

Supplemental Material

## Data Availability

The data supporting this study are not publicly available, but may be provided by the corresponding author upon reasonable request.
