# Supplementary material for: Patients’ experiences of analgesic use for chronic musculoskeletal pain in primary care: An interview study in Germany
Source: Eur J Gen Pract. 2026 Nov 2;32(1):2747452. doi: 10.1080/13814788.2026.2747452 (PMC13757892; doi:10.1080/13814788.2026.2747452)
Supplement: Supplementary file 1 [file IGEN_A_2747452_SM0153.docx]

***Supplementary table 1:Pain medications mentioned by participants.***

*The table provides an overview of the pain medications mentioned by patients during the interviews.*

| **Medication category** | **Medications mentioned by participants** | **Summary of participants’ reports** |
| --- | --- | --- |
| Non-opioid analgesics | Ibuprofen, metamizole, paracetamol,  diclofenac, naproxen, coxibs | Ibuprofen was described as the first-line analgesic, most commonly used as an on-demand medication, with generally positive effects. Gastrointestinal adverse effects were frequently reported and were often managed with proton pump inhibitors, while renal impairment was recognized as a contraindication.  Metamizole was commonly mentioned when pain relief with other non-opioid analgesics was insufficient and was mainly used as long-term therapy, either alone or in combination with other analgesics. It was generally perceived as effective and well tolerated, although isolated adverse events (e.g., elevated liver enzymes, hypotension, anaphylactic shock) were reported. Participants were aware of the risk of agranulocytosis but generally considered its benefits to outweigh this risk.  Paracetamol was reported to play only a minor role in the management of chronic musculoskeletal pain and was mainly used for mild pain, colds, or headaches. Patients were aware of the risk of liver toxicity, and some discontinued treatment because of elevated liver enzyme levels.  Diclofenac, naproxen and coxibs were used by some participants as regular or on-demand medication. Adverse effects, including gastrointestinal symptoms and renal impairment, led to discontinuation in some cases. |
| Weak opioids | Tramadol, tilidine | According to patients’ reports, opioids were predominantly prescribed in exceptional circumstances – such as in cases of acute herniated discs or for severe pain – and were usually only used for short periods. Only a few patients reported long-term opioid therapy; the process of individual dose titration was frequently described as protracted. Some patients also used opioids as on-demand medication. Compared with other analgesics, the most side effects were reported with opioids, including fatigue, nausea, constipation, sweating, tremors, irritability and psychological changes. In addition, inadequate pain relief and experiences of dependence with withdrawal symptoms were described. Patients were aware of the potential for dependence, which often lead to a cautious attitude towards opioid therapy. |
| Strong opioids | Morphine, oxycodone, fentanyl, tapentadol |  |
| Co-analgesics | Antidepressants: Amitriptyline, duloxetine, paroxetine  Anticonvulsants: Gabapentin, pregabalin  Muscle relaxants: methocarbamol, flupirtine | Co-analgesics such as antidepressants and anticonvulsants played a minor role overall in patients’ reports. Opinions on their effectiveness varied: Whilst some patients reported good pain relief, for others the benefits were limited by side effects such as sleep disturbances, restlessness, drowsiness or elevated liver enzymes. The use of muscle relaxants was also described in isolated cases, with reports of poor tolerability in these instances as well. |
| Others | Biologics, glucocorticoids, medical cannabis | Other medicines mentioned included biologics and glucocorticoids for patients with underlying rheumatic conditions, as well as medical cannabis. Experiences with cannabis varied: Whilst some patients reported good pain relief, others described side effects such as nausea or dizziness. Furthermore, medical cannabis was regarded by some as a potential treatment option, although its use is hampered by bureaucratic hurdles and a reluctance to prescribe it. Glucocorticoids were used primarily for rheumatological conditions, with patients being aware of the potential side effects and preferring the lowest possible maintenance dose. |
